# Interpreting surveys to estimate the size of the monarch butterfly population: Pitfalls and prospects

**DOI:** 10.1371/journal.pone.0181245

**Published:** 2017-07-14

**Authors:** John M. Pleasants, Myron P. Zalucki, Karen S. Oberhauser, Lincoln P. Brower, Orley R. Taylor, Wayne E. Thogmartin

**Affiliations:** 1 Department of Ecology, Evolution and Organismal Biology, Iowa State University, Ames, Iowa, United States of America; 2 School of Biological Sciences, The University of Queensland, Brisbane, Australia; 3 Dept. of Fisheries, Wildlife and Conservation Biology, University of Minnesota, St. Paul, Minnesota, United States of America; 4 Sweet Briar College, Sweet Briar, Virginia, United States of America; 5 Department of Ecology and Evolutionary Biology, University of Kansas, Lawrence, Kansas, United States of America; 6 US Geological Survey, La Crosse, Wisconsin, United States of America; University of Southern California, UNITED STATES

## Abstract

To assess the change in the size of the eastern North American monarch butterfly summer population, studies have used long-term data sets of counts of adult butterflies or eggs per milkweed stem. Despite the observed decline in the monarch population as measured at overwintering sites in Mexico, these studies found no decline in summer counts in the Midwest, the core of the summer breeding range, leading to a suggestion that the cause of the monarch population decline is not the loss of Midwest agricultural milkweeds but increased mortality during the fall migration. Using these counts to estimate population size, however, does not account for the shift of monarch activity from agricultural fields to non-agricultural sites over the past 20 years, as a result of the loss of agricultural milkweeds due to the near-ubiquitous use of glyphosate herbicides. We present the counter-hypotheses that the proportion of the monarch population present in non-agricultural habitats, where counts are made, has increased and that counts reflect both population size and the proportion of the population observed. We use data on the historical change in the proportion of milkweeds, and thus monarch activity, in agricultural fields and non-agricultural habitats to show why using counts can produce misleading conclusions about population size. We then separate out the shifting proportion effect from the counts to estimate the population size and show that these corrected summer monarch counts show a decline over time and are correlated with the size of the overwintering population. In addition, we present evidence against the hypothesis of increased mortality during migration. The milkweed limitation hypothesis for monarch decline remains supported and conservation efforts focusing on adding milkweeds to the landscape in the summer breeding region have a sound scientific basis.

## Introduction

The eastern migratory population of the monarch butterfly, *Danaus plexippus*, declined over 80% from 1999 to 2016, as measured by the area (hectares) of trees covered by monarchs at several overwintering sites in Mexico (based on data in Rendon-Salinas et al. [1]). The Eastern population summer breeding range is bordered on the west by the eastern portions of Kansas, Nebraska, South Dakota and North Dakota and extends through the Midwest to the Northeast and north into lower Canada. The Midwest portion of the summer breeding range has been identified using stable isotopes as the major contributor to the overwintering pop [2,3]. Pleasants and Oberhauser [4] and Flockhart et al. [5] made the case that the decline was due to a loss of milkweeds, the sole host plant of monarch butterflies, in Midwest corn and soybean fields due to the use of glyphosate herbicide (the milkweed limitation hypothesis). However, studies based on long-term data sets of butterfly and egg counts in the Midwest have failed to detect a population decline [6–9], leading some of these authors [6–8] to conclude that there has been no decline in the summer population and that the decline seen on the overwintering sites in Mexico must be due to increased mortality during migration.

Knowledge of the factors responsible for the observed decline in the monarch overwintering population can inform conservation efforts, and predict the expected response of the population to habitat restoration. Analyses seeking to identify those factors require reasonably credible measures of population size. Credible measures of population size are also needed to determine the influence of weather, climate and other factors on population size variation. The most commonly cited index of the size of the eastern migratory monarch population is the number of hectares with trees covered by monarchs at the overwintering sites in Mexico [1]. This population size is the result of successive generations beginning with one generation in Texas and Oklahoma that consists of the offspring from adults that have returned from Mexico and two or more generations from adults that have moved north into the summer breeding zone [10]. Estimated indices of population size in each of these generations can inform analyses of factors affecting final population size (e.g. [11]).

Butterfly surveys by citizen scientists during the breeding season and portions of the monarch annual migratory cycle have been used as indices of monarch numbers. For example, annual counts conducted by North American Butterfly Association (NABA) volunteers at sites throughout North America, and state monitoring networks, especially those in Illinois and Ohio [12], have provided data used for several studies investigating factors affecting population size [7–8,11,13]. Similarly, counts of monarch eggs per milkweed stem have been used as an index of population size. The Monarch Larva Monitoring Project (MLMP) [14] obtains data from dozens of citizen scientists monitoring patches of milkweeds weekly for eggs and larvae. Several studies have used the average eggs per stem as an index of population size [9,15]. A variant on this approach is to multiply the average eggs per milkweed stem by the number of milkweed stems available on the landscape [4,16]. It should be noted that all of these methods are indices of population size and not actual estimates of the number of monarch individuals. Even efforts to convert hectares of butterflies in Mexico to actual number of butterflies can do no better than identify a limited range of values for butterflies per hectare [17].

Here, we focus on Midwest surveys of adult butterflies and eggs and larvae per milkweed stem and point out a key flaw associated with the interpretation of survey data by several studies [6–8] that there was no decline in the summer population. The problem with these surveys is that they are conducted in habitats that are natural or semi-natural areas; agricultural fields are not included [18]. However, over the two decades that these surveys were conducted, the milkweeds in agricultural fields have virtually disappeared and the majority of monarch reproductive activity has gone from occurring in agricultural fields to non-agricultural areas [4,16]. We investigate how estimates of population size over time based on surveys in non-agricultural habitats are affected when milkweeds in agricultural fields disappear and monarch activity shifts from agricultural fields to the surrounding non-agricultural areas.

Dyer and Forister [19] modelled a hypothetical monarch population that had shifting activity from Habitat A (agricultural fields) to Habitat B (non-agricultural areas). They concluded that if there was a population decline it could be detected just by looking at the number of individuals in Habitat B. However, if the shift from Habitat A to B took place over a small number of years then detection was more difficult [19]. Although their models indicated that a population decline could be detected, the rate of decline observed in Habitat B was much less than the actual rate of decline used in the model.

Our analysis is based on the premise that on the landscape, at any point in time, the distribution of adult monarchs will mirror the distribution of milkweeds. Empirical support for this premise comes from Kasten et al. [20] who found that adult monarchs were five times more likely to be observed in roadside areas with patches of milkweeds than areas without such patches. In addition, in milkweed patches the number of eggs and larvae per m^2^ increased as the number of milkweed stems per m^2^ increased. Thus, monarchs are more likely to be observed where there are milkweeds and when there are more milkweeds there is likely to be more monarch activity. Female monarchs are drawn to patches of milkweed for egg laying and likely to remain in patches for some time as they choose plants and lay eggs [21–22]. Male monarchs are also drawn to patches which serve as mating arenas and they remain in patches for some time patrolling for females [23]. Of course, some monarchs may be found in areas where there are no milkweeds. Some may be transiting between milkweed patches [12] and some may be drawn to sites with nectar that have no milkweed. However, the probability of finding monarchs during the breeding season will be greater in areas with milkweed than in areas without milkweed.

In this study, we use available data on the change in the proportions of milkweeds in corn and soybean fields and in non-agricultural habitats to show the consequences of using butterfly and egg counts conducted in non-agricultural habitats to estimate changes in total population size. Based on the premise of monarchs co-occurring with milkweeds, we posit that the proportion of monarch activity in each of these habitats will correspond to the proportion of milkweeds on the landscape in each of these habitats. We use data on milkweed densities in different habitats and the land cover of those habitats to estimate the proportion of landscape milkweeds in agricultural and non-agricultural habitats over the last two decades.

Our estimates of how the number of milkweeds in agricultural fields and non-agricultural areas has changed over time allow us to examine counts of adults and eggs and separate the effect on those counts due to the shifting proportions of monarch activity in each of the two habitats from the effect due to population size. We use these corrected counts (with the shifting proportion effect removed) as a better indicator of population size and examine the trend in this indicator over time and the degree of correlation with the size of the overwintering population. It is important to point out that generating these corrected counts from the shifting proportions of milkweeds does not mean that these estimates of population size will necessarily have to decline as the number of milkweeds declines. It is possible that the population size could remain constant if the density of monarchs in the remaining milkweed patches increased sufficiently.

Lastly, we also examine the hypothesis that increased mortality during migration is responsible for the monarch population decline. If the migration mortality hypothesis is correct we would expect that summer butterfly or egg counts would be a progressively poorer predictor of the size of the overwintering population over the last two decades.

## Methods

Our analysis applies to the Eastern population of the monarch butterfly that migrates to Mexico. We focus on the Midwest portion of the breeding range (the North Central region of the monarch range as defined by Stenoien et al. [9] and Oberhauser et al. [24]) because isotope analysis indicates that this is the area from which the majority of butterflies that migrate to Mexico originate [2,3]. Thus what happens in the Midwest will have a major effect on the size of the population [4].

Our analysis is based on estimating the change in the proportion of milkweeds on the landscape in agricultural fields and non-agricultural areas in the Midwest over the last two decades. This estimate requires information on milkweed densities in those two types of habitats and the amount of area covered by each. For milkweed density we only have information beginning in 1999 [25] so, although the analyses of Inamine et al. [6] and Ries et al. [5] begin in 1993, we have begun our analysis in 1999. Milkweed densities are based on state-wide surveys in Iowa conducted in 1999 [25] and 2009 [26] in corn and soybean fields and in non-agricultural habitats such as roadsides and CRP (Conservation Reserve Program) land, but excluding forested land (see S1 Table for non-agricultural habitat categories). Although other estimates of milkweed density exist for specific habitats such as roadsides [20] or natural areas [27], only the Iowa data cover all relevant habitat types, so we have used Iowa data for consistency. The Iowa surveys measured the area covered by milkweeds (m^2^) per hectare. We converted area to milkweed stems/ha using factors of 1.62 stems/m^2^ in corn and soybean fields [28] and 1.95 in non-agricultural areas [5]. Milkweed density in the non-agricultural areas measured in 1999 and 2009 did not change significantly, so we assume that milkweed densities in non-agricultural areas have remained constant, as was also found for non-agricultural areas in Illinois surveyed since 1997 [27]. However, milkweed densities in agricultural fields in Iowa declined 97% between 1999 and 2009 [25,4]. An Illinois study [27] estimated a 94% decline in milkweeds in agricultural fields over this period. To estimate the rate of change in milkweed density in agricultural fields over the years, we use data from milkweed patches within agricultural fields monitored in Iowa from 2000 through 2008. The decline over time fit an exponential decay function [28] so we used an exponential decay function intersecting the 1999 and 2009 survey milkweed density data points to estimate the rate of decline in agricultural field milkweeds [28].

Ideally, we would like to know the total number of milkweed stems in different habitats over time across the Midwest monarch range. However, we only have milkweed density information for Iowa. We assume, as did Pleasants and Oberhauser [4] and Pleasants [16,28], that milkweed densities in the different habitats in Iowa are similar to the densities in those habitats in other parts of the Midwest within the monarch range. Certainly we expect that the change in milkweed density in fields in Iowa, which are dominated by corn and soybeans, is similar to that of agricultural fields throughout the Midwest [27], for which 92% of agricultural land is also in corn and soybean production [29]. In addition, the adoption of herbicide-tolerant varieties of corn and soybeans and the use of glyphosate herbicide has been similar throughout the Midwest to the point where they now comprise >90% of corn and soybean plantings [30].

To estimate the number of milkweeds in agricultural and non-agricultural habitats we combined milkweed density information with information on the area of different milkweed-suitable habitat types using Iowa land use data [29,31–32] (S1 Table). This estimate assumes that land use in Iowa is similar to that of the Midwest monarch range. The proportion of the milkweed-suitable landscape covered by different habitat types is not exactly the same for the Midwest as for Iowa [28]. However, the proportion of milkweeds in agricultural fields in Iowa at the beginning of the sampling period in 1999 is similar to that for the Midwest as a whole (73% vs 63% [28]). As additional support for using Iowa data as a proxy for the Midwest, Pleasants and Oberhauser [4] and Pleasants [16,28] showed that the estimate of the summer population size based on Iowa milkweed abundances in conjunction with egg density data from the Monarch Larva Monitoring Project [14] was a strong predictor of the size of the overwintering population.

To estimate the proportion of monarch activity associated with agricultural and non-agricultural habitats we need to know the relative utilization of the milkweeds in those two types of habitats. To do this, we must account for the fact that, on a per stem basis, there are more eggs per milkweed stem in agricultural fields than in non-agricultural areas. Pleasants and Oberhauser [4] found that over a 4-year period, egg density in agricultural fields was, on average, 3.9 times higher than in non-agricultural areas, suggesting that monarchs will be found in agricultural fields above what would be expected based solely on the number of milkweeds. Thus, to determine the expected amount of monarch activity in agricultural fields, we multiplied the number of milkweeds in this habitat by 3.9 to produce what we refer to as “milkweed resource”; for non-agricultural areas we simply use the number of stems as a measure of milkweed resource. The relative amounts of “milkweed resource” in agricultural and non-agricultural habitats will determine the proportion of expected activity. We have also considered apportionment of activity based on the number of milkweeds alone as a more conservative estimate of the shift in monarch activity.

We examined two scenarios for how butterfly or egg counts outside of agricultural fields would respond as monarch activity in fields decreased. In Scenario 1, we let the overall population remain constant while population activity shifted out of fields into non-agricultural areas, testing the assertion that the summer population has remained relatively constant over the years [6–9]. In Scenario 2, we let the overall population decline at the same rate as the decline in the total milkweed resource while the shift out of fields occurred. Scenario 2 corresponds to the hypothesis that milkweed availability limits monarch population size [4]. For both scenarios we use the proportion of the total milkweed resource found outside of fields as an indicator of the proportion of the monarch population found outside of fields.

Knowing the proportion of monarch activity in agricultural and non-agricultural habitats allows us to correct butterfly or egg counts to better reflect the actual population size. At any point in time *t* the total population *N*_*t*_ is equal to *N*_ag_ + *N*_non-ag_, where*N*_ag_ is the number of butterflies or eggs in agricultural fields and *N*_non-ag_ is the number in non-agricultural habitats. We can use the butterfly or egg counts as an index of *N*_non-ag_. The proportion of the total population in non-agricultural habitats *P*_non-ag_ is equal to *N*_non-ag_ / *N*_*t*_. We estimate *P*_non-ag_ from the number of milkweeds in non-agricultural habitats relative to the total amount of milkweed resource. While this estimate is approximate, given the limitations described above, it is based on biologically reasonable assumptions. Substituting butterfly or egg counts for *N*_non-ag_ we can solve for *N*_*t*_ viz:

$$N_{t}\;=\;N_{\text{non-ag}}/P_{\text{non-ag}}.$$

We can then ask whether this corrected index of population size is correlated with the size of the overwintering population, and whether it declines over time and at the same rate as the decline in the overwintering population.

To test the hypothesis that increased mortality during migration was causing the decline in the size of the overwintering population we compared the corrected butterfly count in each year with the size of that year’s overwintering population. The increased migration mortality hypothesis would predict that the summer population count would be a progressively poorer predictor of the overwintering hectares from 1999 to 2014. Thus, the residuals from the regression of corrected butterfly counts and overwintering hectares should be positively correlated with year (the residuals should be larger for more recent years). As a further test, we examined the rate of change in the summer population numbers with those in the overwintering population. The increased mortality hypothesis would predict that the decline would be steeper for the overwintering population than the summer population. To make this comparison we put both trend lines on the same scale so each data set was normalized to a mean of 0 and a standard deviation of 1.

For displaying regressions, we calculated the 95% confidence interval around the fitted line of each regression. These confidence limits reflect the uncertainty in the relationship between the covariate and response, but not the uncertainty in the calculation of the variable itself. For example, NABA counts are an index of uncertain precision which is not included in the regressions. All calculations were conducted in R 3.3 [33].

For this analysis we used the Midwest NABA data presented by Inamine et al. [8] and for eggs per stem we used Midwest MLMP data [9], with the removal of values from individual citizen scientists that were outliers (>2 standard deviations from the mean in a particular year) and values for agricultural habitats that were collected in only one year (2000) (Table 1). For the size of the population in Mexico, we used overwintering hectares as measured by Rendon et al. [1] with corrections as indicated in Taylor [34] (Table 1). We did not use data from before 1999 because 1999 is the first year for which data are available on the number of milkweeds in agricultural fields and other habitats. It should be noted that milkweed decline in agricultural fields was already underway at this point because glyphosate-tolerant crops were introduced in 1996. In 1999, 56% of soybeans and 8% of corn was glyphosate-tolerant [30].

**Table 1:** Data on Midwest adult monarch butterfly counts, Midwest eggs per milkweed stem and area of the overwintering population in Mexico from 1999 to 2014. The first two measures are presented in their original form and then the corrected version.

	Butterfly counts		Eggs per stem		overwinter
year	NABA^1^	corrected^2^	counts^3^	corrected^4^	hectares^5^
1999	256	1066	0.263	1.097	8.97
2000	150	475	0.136	0.430	2.83
2001	308	742	0.461	1.111	9.36
2002	166	329	0.171	0.339	7.54
2003	193	329	0.244	0.417	8.50
2004	59	88	0.127	0.190	2.19
2005	163	221	0.228	0.310	5.91
2006	338	423	0.360	0.450	6.87
2007	266	313	0.339	0.399	4.61
2008	170	193	0.254	0.288	5.06
2009	185	204	0.194	0.214	1.92
2010	307	330	0.349	0.375	4.02
2011	140	148	0.309	0.325	2.89
2012	170	176	0.218	0.227	1.19
2013	42	43	0.095	0.098	0.67
2014	99	101	0.215	0.220	1.13

^1^ from [8];

^2^ NABA count /non-agricultural proportion;

^3^ from [9];

^4^ eggs per stem count /non-agricultural proportion;

^5^ from [1] with corrections from [34];

## Results

To quantify the effect of sampling done only in non-agricultural areas on population size estimates, we first quantified the shift in the proportion of milkweed, and thus monarch activity, in agricultural and non-agricultural habitats. From 1999 to 2014, the number of milkweeds in agricultural fields in Iowa declined by 99.4% as a result of the use of glyphosate herbicide (Table 2). Non-agricultural milkweeds increased 20% from 1999 to 2007. This resulted from increased enrollments in the Conservation Reserve Program (CRP), which converted cropland into non-agricultural habitat [32]. However, this was followed by a similar percentage decline in non-agricultural milkweeds from 2008 to 2014 due to the reversal of CRP enrollments [32] and the loss of grassland to crops because of the high demand for corn resulting from the Renewable Fuel Standard Program begun in 2007 [35]. Overall, the total number of milkweed stems declined 46% from 1999 to 2014, and because an initial increase in non-agricultural milkweeds was followed by a decrease of similar magnitude, virtually all of this loss occurred in agricultural fields. Accounting for the higher use of agricultural milkweeds by monarchs, the total milkweed resource declined 76% from 1999 to 2014 (Table 2). Table 2 also shows the expected change in the proportion of milkweeds in each of the two types of habitat which corresponds to the change in monarch activity. In 1999, 76% of monarch activity would have been in agricultural fields whereas, by 2007, 85% of monarch activity would have been in non-agricultural areas (Table 2). Therefore, from 1999 to 2007, the proportion of the population observed in areas where NABA butterfly or eggs per stem counts were conducted would have changed dramatically.

**Table 2:** Change in the number of milkweed stems and milkweed resource in agricultural fields and non-agricultural habitats from 1999 to 2014 for Iowa (stems and milkweed resource in millions).

	Agricultural fields		Non-agricultural	Total	Total	Proportion of resource	
year	stems	resource^1^	stems	stems	resource^2^	ag	non-ag
1999	172.0	671	211.1	383	880	0.76	0.24
2000	123.5	482	221.6	345	702	0.69	0.31
2001	87.1	340	240.0	327	579	0.59	0.41
2002	62.2	242	245.7	308	488	0.50	0.50
2003	44.9	175	247.2	292	422	0.42	0.58
2004	32.1	125	248.3	280	373	0.34	0.66
2005	22.9	89	250.4	273	340	0.26	0.74
2006	16.3	64	254.1	270	318	0.20	0.80
2007	11.7	46	256.3	268	302	0.15	0.85
2008	8.5	33	241.7	250	275	0.12	0.88
2009	6.1	24	232.0	238	256	0.09	0.91
2010	4.3	17	225.8	230	243	0.07	0.93
2011	3.1	12	228.0	231	240	0.05	0.95
2012	2.3	9	226.3	229	235	0.04	0.96
2013	1.6	6	215.4	217	221	0.03	0.97
2014	1.1	4	206.9	208	211	0.02	0.98

^1^ Milkweed resource in agricultural fields = agricultural stems × 3.89 (factor by which the eggs/stem exceeds eggs/stem in non-agricultural habitats)

^2^ Total milkweed resource = agricultural milkweed resource + number of non-agricultural stems

**Fig 1:**
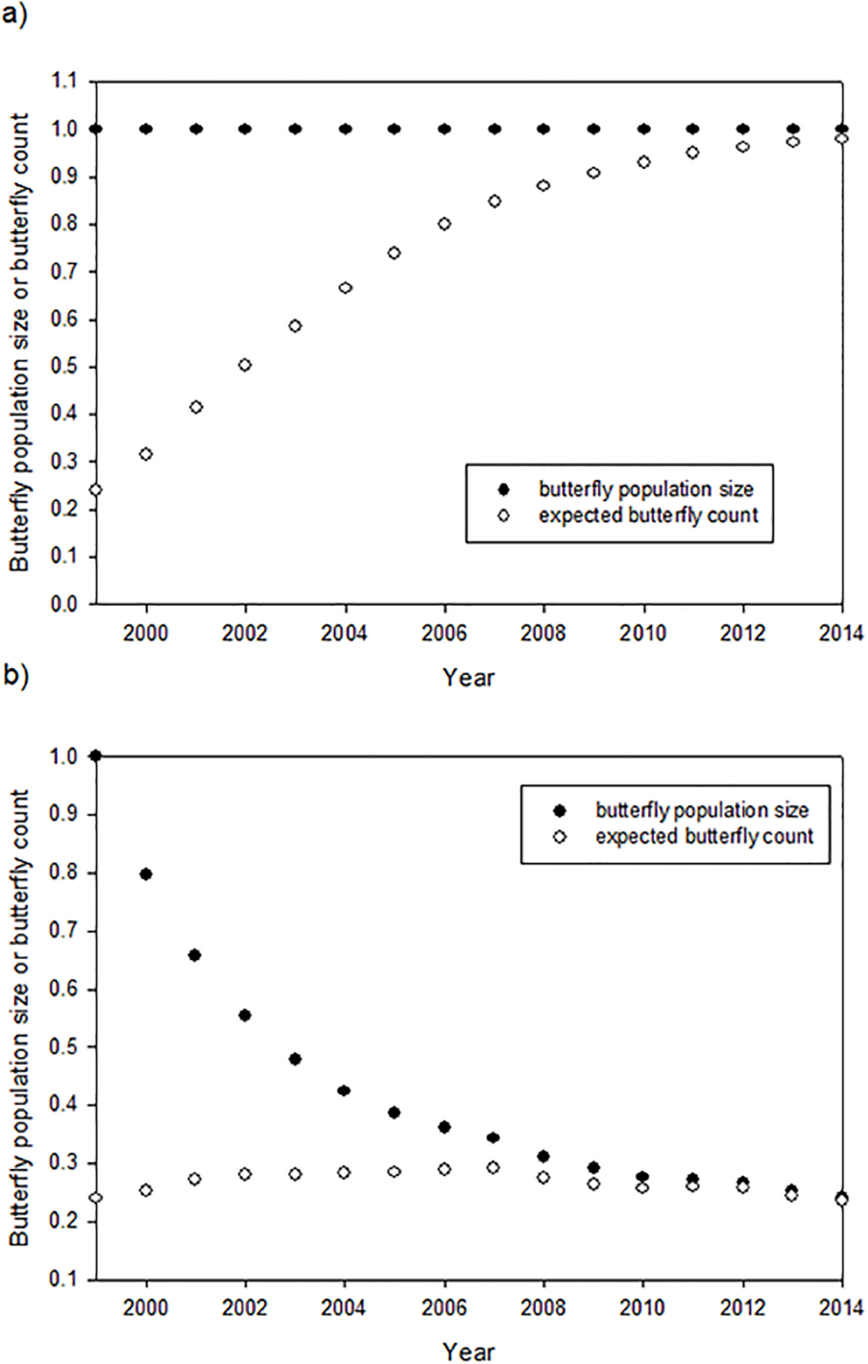
a,b. Expected shape of the relationship between butterfly counts made in non-agricultural habitats through the years under two scenarios. a) Scenario 1: the monarch population is constant from 1999 to 2014; b) Scenario 2: the monarch population is declining along with the decrease in milkweed resources on the landscape. The size of the butterfly population is normalized to begin at 1.0 in each case. For each scenario the expected count at any time is equal to the proportion of the population that is in the non-agricultural habitat times the population size.

The effect of taking butterfly or eggs per stem counts in non-agricultural habitats as an indicator of population size can be appreciated by comparing two scenarios. We focus on butterfly counts, but because both adult butterfly counts and eggs per stem measure monarch activity only in non-agricultural areas we expect them to behave similarly. In fact, the two measures are highly correlated (R^2^ = 0.72, *F*_1,14_ = 38.85, *β* = 0.0095, *p* = 0.00003). In Scenario 1, the monarch summer population remains constant over time as the activity shifts from agricultural to non-agricultural areas. If the summer population was truly constant, as Ries et al. [7] and Inamine et al. [8] asserted, we should see a larger number of butterflies in non-agricultural areas as an increasing fraction of the population shifts to non-agricultural areas (Fig 1a). Neither the NABA nor eggs per stem counts from 1999 to 2014 show an increase over time as expected if the population was constant under Scenario 1 (Table 3, rows 2 and 3). In Scenario 2, the population declines as agricultural milkweeds disappear, along with a shift in activity from agricultural fields to non-agricultural areas (Fig 1b), as would be expected under the milkweed limitation hypothesis. In Scenario 2, counts increase somewhat from 1999 to 2007 and decrease thereafter. If Scenario 2 was actually the case then regressions would show no trend over time. This is what was found by Ries et al.[7] and Inamine et al. [8] (Table 3, rows 2 and 3). They misinterpreted this lack of a trend as indicating no change in population size.

**Table 3:** Regression relationships between variables and year and between variables and overwintering hectares (OW ha).

	Response	Predictor	β	*F*_1,14_	*R*^2^	*p*
1	OW ha	year	-0.465	17.83	0.56	0.0009
2	NABA	year	-6.069	1.74	0.11	0.2080
3	Eggs per stem	year	-0.002	0.18	0.01	0.6800
4	NABA corrected	year	-40.530	16.32	0.54	0.0122
5	Eggs per stem corrected	year	-0.041	11.26	0.45	0.0047
6	OW ha	NABA	0.022	9.42	0.40	0.0083
7	OW ha	NABA corrected	0.008	17.06	0.55	0.0010
8	OW ha	Eggs per stem	16.421	5.80	0.29	0.0304
9	OW ha	Eggs per stem corrected	7.790	19.55	0.58	0.0006
10	OW ha	Log(num milkweed stems)	12.409	15.80	0.53	0.0014
11	OW ha	Log(milkweed resource)	4.635	11.94	0.46	0.0039
12	OW ha	Log(annual production)^1^	4.091	36.77	0.72	<0.0001

^1^ Production in any year = milkweed resource × eggs per stem for that year

**Fig 2:**
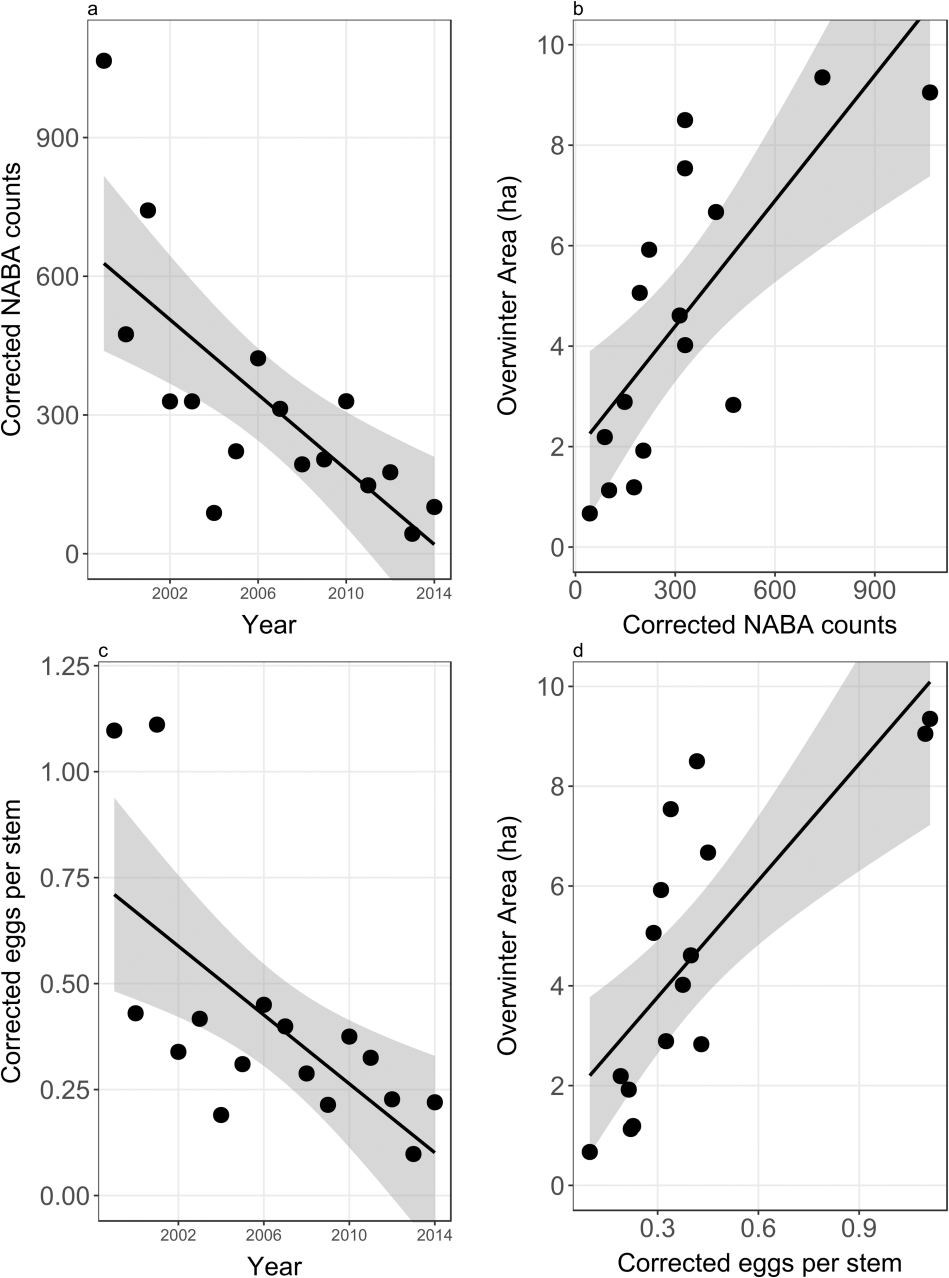
a) Corrected NABA counts vs Year, R^2^ = 0.54, *p* = 0.01; b) Overwintering hectares vs corrected NABA counts R^2^ = 0.55, *p* = 0.001; c) Corrected Eggs per stem vs Year, R^2^ = 0.45, *p* = 0.005; b) Overwintering hectares vs Corrected eggs per stem, R^2^ = 0.58, *p* < 0.001.

Our estimates of the year-to-year change in the proportion of the milkweeds in non-agricultural habitats, allow us to disentangle the contribution to counts due to the observed proportion of the population from that due to the actual size of the population. These corrected counts (Table 1) should better reflect the size of the population. Corrected NABA counts and corrected eggs per stem counts now show a significant negative trend over time (Table 3, rows 4 and 5, and Fig 2a and 2c), mirroring the negative trend in the size of the overwintering population. Corrected NABA counts and corrected eggs per stem counts are significantly positively correlated with the size of the overwintering population (Table 3, rows 7 and 9, Fig 2b and 2d) and the correlation is improved over uncorrected counts (Table 3, rows 6 and 8).

We used the higher density of eggs on stems in agricultural fields as an indicator that the activity in fields was greater than the number of stems there would suggest. However, our results are not dependent on having multiplied the number of agricultural milkweeds by 3.9 to create a measure of “milkweed resource”. If we simply use the number of stems in agricultural and non-agricultural habitats to determine shifting proportions, the corrected NABA counts are still correlated with the size of the overwintering population (R^2^ = 0.58, *F*_1,14_ = 19.43, *β* = 0.019, *p* < 0.001) and show in accordance with the milkweed limitation hypothesis a decreasing trend over the years (R^2^ = 0.35, *F*_1,14_ = 7.68, *β* = -14.9, *p* = 0.015).

**Fig 3:**
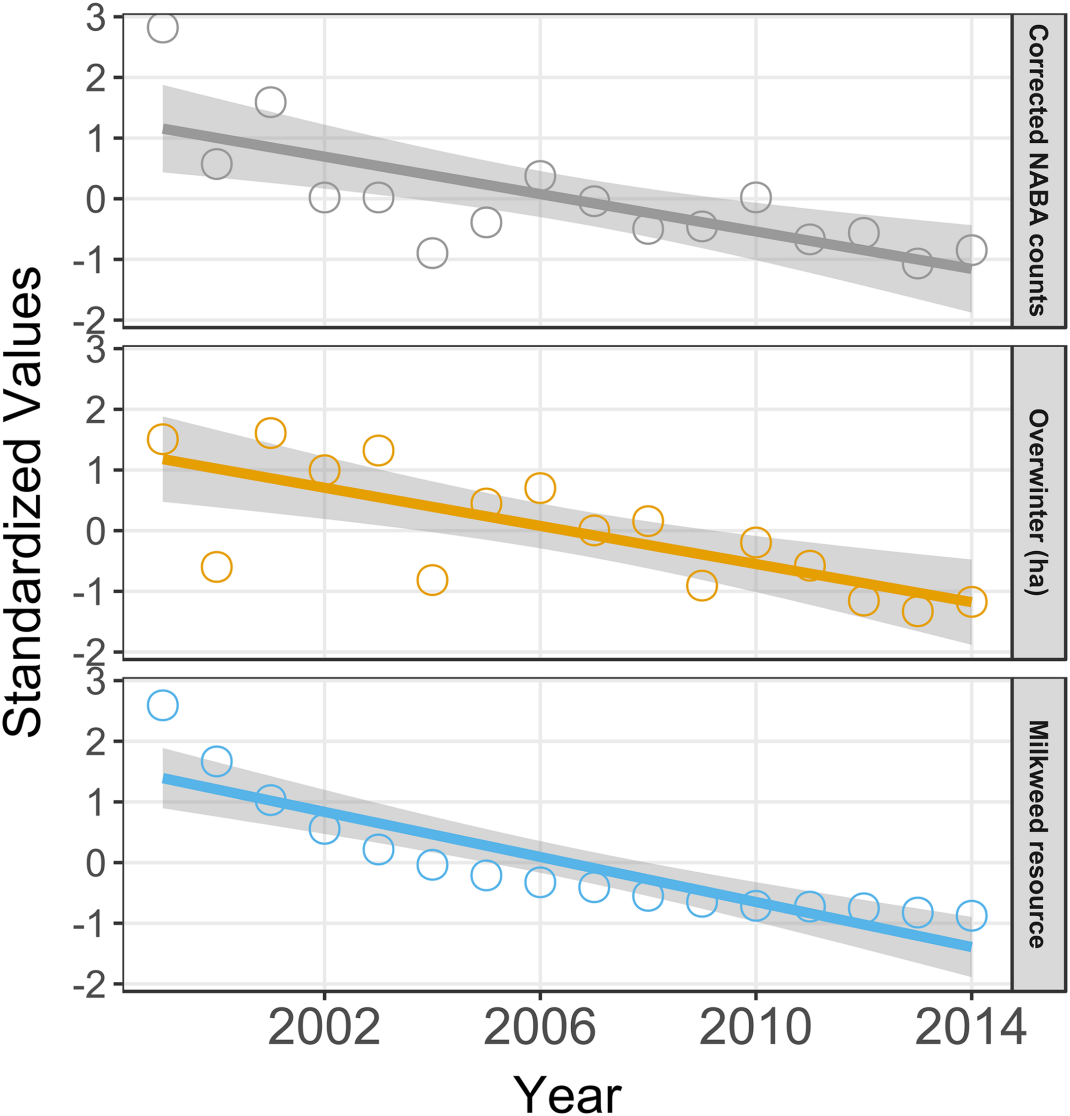
Regression of normalized values of overwintering hectares, corrected NABA counts and milkweed resource vs year. Corrected NABA counts vs year, R^2^ = 0.53, *p* = 0.001, *β* = -0.1541; OW ha vs year, R^2^ = 0.56, *p* < 0.0001, *β* = -0.1572; Milkweed resource vs year, R^2^ = 0.78, *p* < 0.0001, *β* = -0.1856;

To determine the extent to which the decline in the summer population accounts for the decline in the overwintering population we can compare the rates of decline for both. These rates are statistically indistinguishable (Fig 3); the rate for overwintering ha = -0.157 (95% conf. -0.077 to -0.237) and for the corrected NABA counts = -0.154 (95% conf. -0.072 to -0.236). Similarly, the rates of decline for milkweed resource and overwintering hectares are also indistinguishable (Fig 3); milkweed resource decline rate = -0.186 (95% conf. -0.129 to -0.242), lending support to the milkweed limitation hypothesis as the driver of both the summer population and the overwintering population decline. Further corroboration of the milkweed limitation hypothesis comes from the positive relationship between the number of milkweed stems and milkweed resource and overwintering hectares (Table 3, row 10 and 11) and the highly positive relationship between the index of monarch production in each year (milkweed resource × eggs per stem) and overwintering hectares (Table 3, row 12) as was examined by Pleasants and Oberhauser [4].

We can a make a direct test of the Inamine et al. [8] hypothesis that increased mortality during migration is the reason for the decline seen on the overwintering grounds. If there was increased mortality during migration, the size of the summer population should be a progressively poorer predictor of the size of the overwintering population. If we take the corrected NABA counts as an indicator of population size this would predict that the residual variation in the relationship between population size and overwintering hectares should increase by year, with larger residuals for more recent years. However, the residual variation is not correlated with year (R^2^ = 0.09, *F*_1,14_ = 1.43, *p* = 0.25). In addition, the rate of decline in the summer population using corrected NABA counts is identical to the rate of decline in the overwintering population (Fig 3). The increased mortality hypothesis would predict that the decline in the overwintering population should be steeper than the decline in the summer population.

## Discussion

By failing to take into account the loss of milkweeds in agricultural fields and the shift in the proportion of the population present in agricultural fields and non-agricultural areas, adult butterfly counts or counts of eggs per stem in non-agricultural areas alone will arrive at a misleading picture of changes in monarch population size. If the monarch population is actually declining, as documented by data from the overwintering colonies, these counts will first increase then decrease (Scenario 2), thus failing to produce a trend over this entire period and leading to an improper inference that the population has remained constant. Correcting for that shifting proportion produces a population size index that exhibits a downward trend over time and is correlated with overwintering population size.

The loss of milkweeds in agricultural fields was nearly completed by 2006, so counts made from 2007 to 2014 are not as subject to the problem of shifting proportions. In fact, uncorrected NABA counts from 2007–2014 show a decline by year (R^2^ = 0.48, *β* = -24.08, *F*_1,6_ = 5.60, *p* = 0.05) as do uncorrected eggs per stem, although the latter is not significant (R^2^ = 0.31, *β* = -0.019, *F*_1,6_ = 2.72, *p* = 0.15). Ries et al. [7] analyzed Illinois Butterfly Monitoring Network (IBMN) summer butterfly counts and found no trend from 1999 to 2006 but a significant decline from 2006 to 2014. Stenoien et al. [9] examined monarch egg densities in non-agricultural areas and found an increase from 1997 to 2006 and a decrease from 2007 to 2014. Similarly, Saunders et al. [11, 13] showed a decline in monarch numbers in Illinois Butterfly Monitoring Network numbers from 2004–2013, but not from 1994–2003. Although counts since 2007 do not have the problem of shifting proportions there are other issues with these counts, including non-random and non-spatially balanced collection of data [18]. Efforts are currently underway to eliminate these problems by establishing a long-term monitoring program for monarchs using random sampling throughout the range and across the habitat types associated with monarch activity, with regularly scheduled visits to count eggs, larvae and adults.

Not all butterfly or eggs per stem counts require a correction factor. Counts from Texas and Oklahoma in the spring when monarchs returning from Mexico are breeding should be fairly accurate as is. This is because these areas consist mainly of pasture and rangeland and have almost no corn and soybean production [31]. Thus, they have not experienced the loss of milkweeds as seen in Midwest agricultural fields and the consequent shift in activity into non-agricultural areas. Estimates of the size of the Northeast portion of the monarch population based on counts should also be fairly accurate because these areas have minimal corn and soybean production [31].

The correction factors that we use are only an approximation. They are not based on extensive sampling of milkweeds in all habitats over time throughout the Midwest. In the future, we may have better data on milkweed densities in different habitats over a wide geographic area but we will not be able to go back in time and determine the change in milkweed densities in agricultural fields. We have used the best data available to estimate the change in milkweed density in agricultural fields.

The NABA counts and eggs per stem counts, when corrected to remove the shifting proportion effect to better estimate population size, are correlated with the size of the overwintering population and show a decreasing trend over the years as does the overwintering population size. Consequently, there is no need to invoke a possible post-summer mortality factor to explain a disconnect between summer and winter population sizes; there is no disconnect. Furthermore, the hypothesis that was raised to account for the apparent disconnect, namely increased mortality during migration, is not supported.

The significant positive relationship between overwintering hectares and three measures of milkweed availability, namely, the number of milkweed stems, milkweed resource, and annual egg production (Table 3) provides direct evidence that the declining availability of milkweed resources is the driver of the decline in the size of the overwintering population [4,5]. It is not surprising that the loss of milkweeds has had an effect on the monarch population; indeed, it would be surprising if the loss of 46% of the milkweeds on the landscape, or 76% of the milkweed resource, did not have an effect. It is important to understand the mechanism by which milkweed resource limitation affects population size. The classic model of how diminishing resources influences population size involves density-dependent mortality with the crowding of individuals into the remaining habitat. While there is lower larval survivorship with higher egg density [5,36] the magnitude of this effect is not sufficient to account for the population decline [36]. More importantly, eggs per stem has not increased over time [9] meaning that higher egg densities and higher larval mortality cannot be responsible for the population decline. Instead, it is hypothesized that the effect of milkweed limitation on population size is mediated through reduced fecundity of monarch females that must find patches of milkweed in an increasingly milkweed-barren landscape. It has been estimated that at least a billion milkweed stems have been lost since 1999, mostly from herbicide use in fields but some from land conversion to crops and development [28]. Not only has the number of milkweeds been reduced on the landscape, the distribution of milkweeds on the landscape has changed [37]. Fifty percent of agricultural fields once had milkweeds [26]; these are now milkweed deserts resulting in an estimated 23.5 million hectares (58 million acres) devoid of milkweeds [28]. Zalucki and Lammers [38] and Zalucki et al. [39] provide models for how these empty areas within the milkweed landscape increase female search time and decrease female monarch realized fecundity, thus reducing the support capacity of the available milkweeds.

The conclusion that the summer population has not changed based on butterfly counts [6–8] led these authors to suggest that conservation efforts should be redirected from putting milkweeds on the landscape to providing resources for migrating monarchs. We do not dispute the need to engage in conservation efforts during all phases of the migratory cycle of monarchs (see also [24]), but our analysis shows that milkweed limitation during summer breeding is the major driver of monarch decline and there is no evidence that there has been increasing mortality during migration. Thus, at a time when federal and state agencies and local groups and individuals are making major efforts to plant milkweeds, it is imperative that the science behind these efforts be clear. The results presented here provide evidence that such restoration efforts are the best way to increase the size of the monarch population.

## Supporting information

S1 Table Milkweed abundance in different habitats in Iowa 1999–2014. Hectares and stems in millions.
(PDF)
